# The attitudes of owners and veterinary professionals in the United Kingdom to the risk of adverse events associated with using non-steroidal anti-inflammatory drugs (NSAIDs) to treat dogs with osteoarthritis

**DOI:** 10.1016/j.prevetmed.2016.07.017

**Published:** 2016-09-01

**Authors:** Zoe Belshaw, Lucy Asher, Rachel S. Dean

**Affiliations:** Centre for Evidence-based Veterinary Medicine, School of Veterinary Medicine and Science, University of Nottingham, Sutton Bonington Campus, Loughborough, Leicestershire, LE12 5RD UK

**Keywords:** Non-steroidal anti-inflammatory drug, Adverse event, Risk, Qualitative, Dog, Osteoarthritis

## Abstract

Non-steroidal anti-inflammatory drugs (NSAIDs) are commonly prescribed by veterinary surgeons for the treatment of canine osteoarthritis, and affected dogs may receive these drugs for long periods of time. Whilst short term administration of NSAIDs to dogs is linked to adverse events such as gastrointestinal haemorrhage and renal injury, reports of adverse events associated with their long-term administration are limited in the veterinary literature. This study aimed to investigate the attitudes towards the long term use of NSAIDs for canine osteoarthritis held by three groups who manage osteoarthritic dogs in the United Kingdom: dog owners, veterinary surgeons and veterinary nurses. A qualitative methodology was adopted, using semi-structured interviews and focus groups. Thematic analysis of these data identified three themes: awareness of potential risks; recognition of adverse events; and influence of risk perception on the use of NSAIDs. Awareness of, and concern about, the risk of adverse events associated with NSAID administration to dogs with osteoarthritis was high in all groups, with veterinary surgeons being one of a variety of information sources used by owners to acquire this knowledge. Veterinary surgeons described difficulty in recognising, managing and avoiding adverse events associated with NSAIDs. When adverse events occurred, a wide range of management approaches were adopted ranging from a brief drug respite to permanent cessation of administration of any NSAIDs to that dog. Commonly employed approaches to minimise risk included dose reduction and screening blood tests. This study describes a high level of concern about the risks associated with long term NSAID administration to dogs with osteoarthritis and highlights a diverse range of strategies employed to minimise these risks. The evidence base for these strategies is poor, and this may present a risk to animal welfare if the affected dogs are not receiving adequate analgesia. In order to address this, more accurate and comprehensive data must be supplied to both veterinary professionals and owners on the true frequency of adverse events associated with long term administration of veterinary NSAIDs and how best to avoid them.

## Highlights

• Many dogs with osteoarthritis receive NSAIDs for long time periods.
• The risk of NSAID-related adverse events concerns veterinary surgeons and dog owners.
• Many strategies are adopted to minimise the risk of these adverse events.
• These strategies may compromise animal welfare through inadequate analgesia.
• The evidence base to support these strategies is inadequate and should be addressed.

## 1 Introduction

Non-steroidal anti-inflammatory drugs (NSAIDs) are commonly used to treat dogs with osteoarthritis (Sanderson et al., 2009). Systematic reviews have assessed the efficacy (Aragon et al., 2007, Sanderson et al., 2009) and safety of veterinary NSAIDs (Innes et al., 2010, Monteiro-Steagall et al., 2013). However, studies reporting long term safety are lacking (Innes et al., 2010), and reporting of adverse events may be incomplete, both in clinical trials and passive surveillance (Hunt et al., 2015). Concern about adverse events associated with the administration of NSAIDs to small animals remains a barrier to their prescription by veterinary surgeons (Bell et al., 2014). The attitudes of pet owners regarding NSAID safety have not previously been reported in peer reviewed literature, but survey data has found a link between safety concerns and poor compliance (Zoetis Inc., 2013). This may have serious implications for the welfare of animals not receiving prescribed analgesia.

The attitudes of patients (Carnes et al., 2008, Milder et al., 2011a, Laba et al., 2013) and healthcare professionals (Mikhail et al., 2007, Cavazos et al., 2008, Braund and Abbott, 2011) to the use and safety of NSAIDs for human osteoarthritis are well documented. Several studies have demonstrated an important role for doctors in educating patients about potential adverse events associated with NSAIDs (Mikhail et al., 2007, Schmitt et al., 2011). The prescribing practice of healthcare professionals is directly influenced by their experiences, leading to avoidance of treatments that have caused adverse events in their own patients (Gabbay and May, 2004, Cavazos et al., 2008). Conversely, patients reliant on NSAIDs for analgesia may ignore the risks if they have not personally experienced an adverse event (Milder et al., 2011b), though perceived risks have been associated with the decision to discontinue NSAID treatment (Laba et al., 2013).

Qualitative research uses a range of approaches including interviews and focus groups to explore motivations, attitudes and opinions (Bryman, 2012). A qualitative approach is particularly useful in areas where little previous research has been conducted and results can be used to inform subsequent quantitative studies (e.g. Wiseman-Orr et al., 2004). Analysis of data derived by qualitative research takes many forms. Thematic analysis (described by Braun and Clarke, 2006) is widely used in healthcare research due to its flexible methodology. The process is slow and iterative with transcribed lines of text coded for their meaning, with codes then collated into larger themes. The reports generated do not feature numbers but instead provide an analytic narrative which makes an argument related to the research question. A growing body of literature derived from qualitative research exists in veterinary medicine (e.g. Coyne et al., 2014, Horseman et al., 2014, Page-Jones and Abbey, 2015).

The aim of this study was to use a qualitative approach to characterise the attitudes of dog owners, veterinary surgeons and veterinary nurses in England and Scotland towards the safety of NSAIDs used to treat osteoarthritis in dogs. The objective was to perform in-depth qualitative interviews with dog owners and focus groups with veterinary surgeons and veterinary nurses to capture a wide range of experiences of using NSAIDs to treat dogs with osteoarthritis.

## 2 Materials and methods

This work forms a part of a larger qualitative study exploring the experiences of dog owners, veterinary surgeons and veterinary nurses in the United Kingdom managing dogs with osteoarthritis. Only results relevant to the aims of this study will be reported. Data were collected using semi-structured interviews for dog owners and focus groups for veterinary professionals (veterinary surgeons and nurses). Ethical approval for the study was granted by the ethics committee at the School of Veterinary Medicine and Science, University of Nottingham. Reporting follows the COREQ checklist (Tong et al., 2007).

### 2.1 Owner interviews

Interviews were conducted between February and August 2014. The inclusion criteria for interviewees were: a) ownership of a dog currently treated or managed for osteoarthritis affecting at least one limb due to any underlying aetiology; AND b) ownership of a dog at least five years of age at the time of the interview; AND c) the owner(s) and dog must live in the United Kingdom. Recruitment was based on a purposive sampling frame constructed by the authors (available on request) containing both dog and owner variables to capture the widest possible range of experiences.

Most interviewees were recruited by placing posters in the waiting rooms of a convenience sample of 10 veterinary practices in England and Scotland. All practices had previously agreed to collaborate with the Centre for Evidence-based Veterinary Medicine, University of Nottingham. Posters asked owners of older dogs with osteoarthritis to contact the lead author by email or telephone to share their experiences. Additional interviewees were recruited by snowball sampling or through the authors’ networks. Owners who expressed an interest in participation were sent information about the purpose of the study including details of the interviewer’s background as a veterinary surgeon and owner of a dog with osteoarthritis. If they agreed to participate, an interview date was then arranged by email or telephone. Incentives to participate were not provided. Interviews were conducted in the owners’ homes by one researcher (ZB) who had received training in qualitative research from the Health Experiences Research Group, University of Oxford. A semi-structured interview guide (available on request), piloted before use, was used to explore owner experiences. Pertinent to this manuscript, interviewees were asked about their experiences of treating their dog’s osteoarthritis, and any previous experience of osteoarthritis in humans or other species. Where owners had more than one dog with osteoarthritis, they chose either to focus on one dog or described their experiences with more than one dog. Interviews were conducted until data saturation was reached (see below).

### 2.2 Veterinary focus groups

Focus groups were conducted between August and December 2014. The inclusion criterion was any practice from which owners had been recruited; veterinary nurses and veterinary surgeons that performed consultations with owners of dogs with osteoarthritis within that practice were then invited to participate. A purposive sampling frame ensured inclusion of a range of practice sizes, locations and types. Meetings were arranged through contact with one member of staff at the practice who then recruited others to participate; attendance was voluntary. Details of the purpose of the study were available in advance. Food was provided as an incentive to attend meetings and to ease discussion (Braun and Clarke, 2013). Focus groups were conducted on the practice premises for convenience and to provide a safe setting for open discussion. The focus group for veterinary nurses was conducted at a different time to that of the veterinary surgeons within the same practice to ensure both groups were comfortable to describe their experiences. All focus groups were conducted by one researcher (ZB). A semi-structured question list was used to prompt the participants but where possible discussion was allowed to proceed with minimal interruption. Questions focused on the diagnosis and treatment of osteoarthritis in dogs.

### 2.3 Thematic analysis

Contextual field notes were made during the interviews and focus groups. Interviews and focus groups were audio recorded and professionally transcribed verbatim. Transcripts were not returned to the participants. Transcribed interviews and focus groups were read and reviewed several times by the lead author. Thematic analysis was performed following the six step plan described by Braun and Clarke (2006) using the organisational support of nVivo (nVivo v10, QSR). Constant comparison was used to ensure all opinions were included (Braun and Clarke, 2006, Coyne et al., 2014). Thematic analysis was performed in tandem with data collection. Data saturation for interviews and focus groups was defined as the point at which no additional themes emerged as a result of analysing new transcripts; at this point recruitment for further participants was halted. For the purpose of this secondary analysis, all extracts from both interviews and focus groups that described use of NSAIDs were scrutinised, and coded into anticipated and emergent themes, as described by Ziebland et al. (2004). Statistical analysis was not performed as the qualitative purposive sampling methodology aimed to capture a wide range of experiences rather than to represent a population (Ziebland et al., 2004, Silverman, 2013).

## 3 Results

Forty-nine owners expressed interest in the study. Seventeen were either ineligible to participate, declined participation when provided with study details or did not have the time to be interviewed during the study period. Thirty-two interviews were conducted, capturing the views of 40 owners about 35 dogs with osteoarthritis treated at seventeen different veterinary practices. Interviews ranged from 52 to 170 min in length. All dogs discussed by owners had received at least one of five different NSAIDs (carprofen, cimicoxib, firocoxib, meloxicam, robenacoxib) for their osteoarthritis. Five focus groups, each of approximately 60 min length, were run in four veterinary practices totalling 31 participants. Four focus groups were conducted with veterinary surgeons. A single focus group was conducted with veterinary nurses in the only practice providing nurse clinics for dogs with osteoarthritis. Thematic analysis of the coded excerpts relating the use of NSAIDs identified three themes: awareness of potential risks; recognition of adverse events; and the influence of risk perception on the use of NSAIDs. Due to the word limits of this publication, exemplary quotations are provided to illustrate each theme.

### 3.1 Awareness of potential risks

Almost all owners were aware of one or more “side effects” associated with the use of NSAIDs in dogs. Many drew analogies between NSAIDs used in humans and those prescribed for their dog. Rarely, owners had personal experience of adverse events due to NSAIDs in people such as stomach ulceration. A few owners had been told about adverse events associated with NSAID affecting the dogs of friends or colleagues, and a couple had experienced adverse events with previous dogs. Most frequently, owners expressed concern about NSAID administration causing “organ damage” and less often gastrointestinal injury including vomiting, diarrhoea and ulceration. Some owners identified specific organs such as the liver or kidneys which could be harmed. Adverse events were thought to be associated with both longevity of dosing and high drug doses.

Some owners recalled warnings provided by the veterinary surgeon at the time of initial NSAID administration. Often these included a comparison with human NSAIDs, which may have assumed that owners would have knowledge of their associated adverse events, and advice to give the medication with food to avoid stomach ulcers. Very rarely, owners recalled being provided with printed information. A few owners asked the veterinary surgeon about the potential harms if no details were given. Drug data sheets were used as a source of information by a couple of owners; others remarked that the data sheet was not always available. One owner highlighted the importance of this:

*Now, the vet didn't tell me this, but I always read the leaflet anyway, and it said with this one [robenacoxib] that it's better to wait at least thirty minutes after food before you give the tablet.* [Owner 5]

The internet was a source of information about NSAIDs for many owners. Most described performing a search using Google using terms such as “dog arthritis” to look for information about treatment options. A few performed a more focused search to look for information about adverse events associated with particular prescription treatments. Their motivation in all cases was to seek information to supplement that provided by their veterinary surgeon. Several owners described finding websites which alarmed them, particularly regarding reports of deaths associated with carprofen. A few described their shock that a veterinary surgeon would prescribe a drug associated with death, though many were aware that they should not trust everything they read on the internet:

*The vets didn't tell me anything about it, and I'm afraid I looked it up on the internet, and found that Rimadyl does disagree with a lot of dogs. There's an awful lot of nasty stories out there about poisoning and all the rest but fortunately I'm well aware of internet world.* [Owner 11]

A few owners described the newer generation NSAIDs as safer based on fewer reports of adverse events on the internet; for example, one owner who was very concerned about carprofen thought cimicoxib had no potential for harm. Several owners said they would appreciate more guidance from their veterinary surgeon about how to interpret what they read online or which sites to trust. Many said they would always want to check what they read on the internet with their veterinary surgeon. Surprisingly, a couple of owners were unaware of any adverse events associated with the drugs, emphasising the important role of the veterinary surgeon in educating owners:

*No, I'm not aware of any side effects [associated with carprofen]. I think if there were side effects [my vets] would tell me so I tend not to Google dogs.* [Owner 30]

All veterinary professionals were aware of the potential for adverse events with NSAIDs; concerns about hepatic and renal compromise and gastrointestinal ulceration were commonly mentioned. Many veterinary surgeons discussed the safety advice they gave to owners; typically, this matched what the owners described. Most veterinary surgeons and veterinary nurses commented that time pressure in the osteoarthritis consultation was a barrier to a longer discussion. This was compounded by many diagnoses of osteoarthritis being made during a consultation for another problem. Some veterinary professionals thought most of their owners were aware of the risks associated with NSAID administration, whilst others were less sure:

*I don't think many of them know in advance. … I don't think in reality before you start them on these things, I don't think many clients have much of an inkling as to the potential side effects* [Veterinary surgeon 1, Focus Group 2]

*I think a lot of owners are aware that, particularly the well-known Metacam, a lot of people are aware that it can be detrimental to the health.* [Veterinary nurse 3, Focus Group 4]

### 3.2 Recognition of adverse events

Some owners reported that their dog had experienced one or more episodes of gastroenteritis whilst receiving an NSAID. Typically this involved vomiting with or without blood, and/or diarrhoea containing fresh blood or melena. Often, gastroenteritis occurred soon after treatment inception but some dogs experienced signs after prolonged NSAID dosing. Some dogs experienced a single episode of vomiting or diarrhoea whilst others exhibited clinical signs for longer.

*[My vet] said ‘Let's try Previcox.' and then she was then having problems with it, she was bringing up frothy stuff, but it had little streaks of blood in it.* [Owner 15]

Owners reported that gastroprotectants such as ranitidine and sucralfate were commonly prescribed after such episodes, and sometimes their use was long-term if NSAIDs were continued. Once signs had abated, some dogs were re-introduced to the same NSAID and experienced no further adverse events, whilst others were switched to a different NSAID or an alternative analgesic such as tramadol. This appeared to be determined by the individual approach of the veterinary surgeon in charge of that case. Only a couple of dogs experienced haematemesis on multiple different NSAIDs; most dogs appeared to tolerate a different NSAID much better.

*She started on Previcox. And that gave her a bleed … Yeah, her poos came all suddenly full of blood. I stopped it immediately, took her back [to the vets], we gave her a break, and then changed her to Rimadyl, and she's been okay on that ever since.* [Owner 16]

Other adverse events associated by owners with NSAIDs were reported much less frequently. No owners reported renal or hepatic compromise as a result of the drugs, though one dog was investigated for hepatic side effects as a result of increasing liver enzymes on routine biochemistry.

*About three months later, because they're monitoring her regularly, [her liver values had] gone down again, then they went up again, then they'd gone down again, so we'd decided that she's just erratic. But it was then the vet said “Oh dear, we'd better have a biopsy.” and all the rest of it. Ooh! Panic! It was very expensive… I'm glad we did it because we ruled out she hadn't got cancer or anything. But I still don't know whether it was the Rimadyl that pushed them up a bit.* [Owner 7]

Veterinary professionals disagreed both within and between practices about the incidence of adverse events, with the veterinary nurses perceiving the incidence to be highest. However, most agreed that adverse events were typically associated with post-operative NSAID use rather than with dogs receiving NSAIDs for osteoarthritis.

*I think it’s one of these where there is no real number. The clients say to you, “What is the risk of vomiting and diarrhoea?” And normally I say about one in 40. I* don’t *think it*’s *any less frequent than that because I think it’s one of those things where probably each of us will diagnose it, to some extent or another, at least once a week.* [Veterinary surgeon 1, Focus Group 1]

*I think sometimes we're attributing [gastrointestinal] signs to the non-steroidals and they're probably not purely caused by non-steroidals. The number of animals that we see with [gastrointestinal] upsets, which look like non-steroidal side effects, where they haven't taken any, is probably bigger than the ones we see that do. So, but obviously you have to err on the side of caution, you have to assume it is the medication that could cause the side effects….* [Veterinary surgeon 3, Focus Group 2]

It was apparent that reporting of adverse events associated with NSAIDs to the Veterinary Medicine Directorate was extremely uncommon, with difficultly recognising true adverse events being one reason for this. Many veterinary surgeons associated adverse events with poor owner compliance, citing examples of incorrect dosing, the drugs being given without food or continued administration to an ill dog as common causes. No veterinary surgeons could recall a case of severe hepatic or renal toxicity related to NSAIDs.

### 3.3 Influence of risk perception on the use of NSAIDs

There was a clear impact on owners and veterinary professionals of the awareness of potential adverse events related to NSAID administration. Many strategies were employed to minimise risks. Several owners stated that their dog should or could not be treated with NSAIDs. Typically this was based on the dog having experienced an adverse event whilst receiving an NSAID. Most veterinary surgeons described their frustration at being unable to persuade the owners of some dogs with osteoarthritis to ever use NSAID treatments. A few owners held extremely strong beliefs about the association between adverse events and specific NSAIDs.

*I'm absolutely anti Rimadyl. It's on my notes that never, ever give my dogs Rimadyl. And then when I find they’ve had Rimadyl I go mad. My parents' cocker spaniel died of platelet eruption on Rimadyl, at seven… It might be good pain relief but I will not have it*. [Owner 3]

Some veterinary surgeons elected to avoid NSAIDs in animals they perceived to be at high risk of adverse events, typically due to slight abnormalities on blood tests. The alternative treatment prescribed was usually tramadol, though several veterinary surgeons questioned its evidence base as an analgesic for canine chronic pain:

*I don’t know what the studies are about what to commonly do, but if anything I think as a practice we’re quite overly cautious I think about [using NSAIDs] − which is a good thing. Sometimes I think am I being overly cautious by starting on tramadol instead of on Loxicom or something; I’m not too sure. [Veterinary surgeon 5, Focus Group 1]*

Several veterinary surgeons and owners described certain NSAIDs as being “safer” than others; cimicoxib, firocoxib and robenacoxib were typically mentioned in this context. A few veterinary surgeons used one of these drugs as a first line for safety reasons, whilst others switched if a dog experienced something believed to be an adverse event. The subjective nature of these decisions was frequently clear when examples were discussed:

*… if [gastroenteritis] came on almost instantaneously, within days of starting carprofen, and the animal was really ill, then I would be really ‘Maybe we can‘t really use non-steroidals with this.' But if on the other hand it came on after a few weeks, and it wasn't particularly bad, then yeah, take it off the carprofen for a while, give it a wash-out period, get his tummy back to being normal and then try on Previcox or Cimalgex. [Veterinary surgeon 1, Focus Group 2]*

Many owners and veterinary surgeons described their desire to reduce the dose of NSAIDs. Some owners asked their veterinary surgeon to reduce the dose and many veterinary surgeons routinely lowered the daily dose over time. Veterinary surgeons often expressed concern about owners taking NSAID dose reduction decisions into their own hands. This was in sharp contrast to their attitude to other treatments such as tramadol and supplements where veterinary surgeons were typically happy for owners to modify the dose. A less common strategy was to reduce dosing frequency to alternate days. Most veterinary surgeons expressed uncertainty about whether, when and how to reduce the dose; within a practice there were frequently several methods. Many acknowledged that they did not know how best to proceed but most seemed confident that lower doses were effective.

*A lot of them do seem fine…if you've got a thirty-kilo dog, they seem quite happy on a twenty-kilo dose. And the owners see all the benefit from it…* [Veterinary surgeon 2, Focus Group 5]

Some owners reported that their dogs underwent regular screening blood tests whilst on NSAIDs; others could not recall their dog ever having been tested. Blood tests were described by owners as both providing comfort and as a source of major concern. One owner, interviewed whilst waiting for blood test results, described how her worry about adverse events had led to a blood test and her subsequent concern about what the results might mean:

*I was getting a bit twitchy… Because now he has Onsior forties every day now…. We did a blood test on Thursday, we won't get the results back til Monday, to see how his insides are coping with his meds. And if they're compromised I'm not quite sure where we go from there.* [Owner 14]

Almost all veterinary surgeons reported that they performed routine blood tests on dogs receiving NSAIDs. The frequency varied widely within and between practices with some veterinary surgeons insisting that dogs received a blood test before starting treatment and others happy to wait several months before testing. Most veterinary surgeons made decisions on the basis of basic blood biochemistry; liver function assessment by bile acid stimulation was not described. Several veterinary surgeons acknowledged that the results of blood tests rarely altered the dog’s treatment, and a few were unsure of how often tests should be performed:

*Personally I think it*’*s the right thing to do the bloods, and I do do the bloods, but whether or not we do it for the right reasons or whether or not there*’*s much evidence that it*’*s actually necessary − I think this is where there is a lack of knowledge around it. I totally recommend people do do them and I do them longer term; but often it*’*s not going to change what I do. More often it*’*s just a monitoring thing.* [Veterinary surgeon 2, Focus Group 1]

Veterinary surgeons recognised the need to balance quality with quantity of life when treating osteoarthritis, though several favoured treatments they perceived to be safer over ones known to be effective in older patients. Despite the risks, most owners continued to give their dog an NSAID, albeit often at a low dose. Many owners described the challenges of trading off pain relief and happiness with the risk of side effects. Opinions varied on striking the right balance:

*So as far as I'm concerned, if he's comfortable I'd rather him be comfortable, and die of liver failure at the age of ten than be in pain but live ‘til twelve*. [Owner 20]

*It would be nice to have eventually painkillers that would not have any side effects. … That would be fantastic, rather than always think ‘Yeah, well, put up with being a bit stiff because I'm worried about painkillers'* [Owner 16]

## 4 Discussion

This qualitative analysis of data derived by interviews with dog owners and focus groups with veterinary professionals demonstrates high awareness and concern about potential adverse events associated with veterinary NSAIDs. This study demonstrates the potential this concern has to affect both prescription of NSAIDs by veterinary surgeons and compliance by owners. It highlights several major issues. Owners not satisfied with the information provided by their veterinary surgeon seek additional guidance, but trustworthy sources are difficult to find. Veterinary surgeons are unsure of the true incidence of adverse events associated with NSAIDs because they are hard to differentiate from concurrent disease. Veterinary surgeons are aware of little evidence to support their decisions to reduce the dose of NSAIDs or to perform regular routine blood tests.

Some owners did not recollect a warning from their veterinary surgeon about the potential for adverse events associated with NSAIDs, and others did not think the information provided was adequate. Based on the focus groups data, it is likely that many veterinary surgeons do warn owners about at least some of the potential adverse events, though many commented on significant time pressures in the consulting room. Concerns about the length (Everitt et al., 2013, Robinson et al., 2014) and complexity (Robinson et al., 2015) of veterinary consultations have previously been raised and are likely to contribute to owners being unable to recall important information. Similar concerns have been raised by doctors prescribing NSAIDs (Mikhail et al., 2007). Many owners turned to the internet to supplement their knowledge. Most who did this recalled using broad search terms which brought up many websites. Little research has been conducted into the use of the internet by pet owners (Kogan et al., 2008) though veterinary surgeons in the United Kingdom believe it to be widespread and detrimental to pet health (British Veterinary Association, 2014). This study confirms the conclusions of Kogan et al. (2008) that owners would prefer to receive information from their veterinary surgeon and would like more advice on which websites to trust.

Many owners of dogs with osteoarthritis expressed concern about the safety of NSAIDs and some preferred to risk efficacy by under-dosing their dog rather than risk adverse events. This is in contrast to the use of NSAIDs in adult human patients with osteoarthritis where qualitative research typically finds patients are unaware of or do not consider the risks (Milder et al., 2011a, Milder et al., 2011b, Schmitt et al., 2011). The decision making role of dog owners is similar to that of parents of young children. Research into attitudes surrounding the risks of childhood vaccination (Wroe et al., 2005) discovered that some parents viewed harms that occurred as a result of a decision not to immunise to be more acceptable than those associated with adverse drug events from vaccination. It is probable that a similar phenomenon occurs in dog owners. This is an area which warrants further research as it has clear implications for compliance and animal welfare as dogs in pain go untreated.

Hunt et al. (2015) report on the frequency of adverse events following NSAID administration reported to the Veterinary Medicines Directorate. It found emesis was the most frequently reported adverse event associated with oral NSAIDs in dogs. Renal and hepatic insufficiently was reported at a low frequency. In our focus groups, some veterinary surgeons linked uncertainty about the incidence of adverse events to their cautious behaviour regarding dosing and monitoring. This highlights the importance of the publication and dissemination of accurate data reporting the incidence of adverse events associated with veterinary medicines. Hunt et al. (2015) recognised that the reported frequency of emesis does not match that of clinical studies. One cause of under-reporting identified in the current study was difficulty in determining the significance of gastroenteritis in dogs receiving NSAIDSs. Clearer guidance may be required on reporting of suspect adverse events to the Veterinary Medicines Directorate.

Several veterinary surgeons commented on the poor evidence base for reduced dosing or monitoring of NSAIDs. Little has been published on either subject in veterinary medicine. A study by Wernham et al. (2011) examined the efficacy of meloxicam in dogs with osteoarthritis at a progressively reduced dose. Many dogs dropped out of the study as their owners perceived analgesia was inadequate but the authors concluded that dose reduction may be effective for some individuals. Use of the lowest effective dose is in dogs advocated by some authors for safety reasons (Lomas and Grauer, 2015) and is advised for doctors using NSAIDs in humans with osteoarthritis (Zhang et al., 2008). More research is urgently needed into the efficacy of low-dose NSAIDs in dogs with naturally occurring osteoarthritis. Many veterinarians performed regular blood tests on dogs receiving NSAIDs but specific guidance on monitoring of hepatic and renal parameters are rarely provided in companion animal NSAID datasheets (National Office for Animal Health (NOAH), 2014). Given the low frequency of renal and hepatotoxicity (Hunt et al., 2015) and the potential costs and morbidity associated with phlebotomy, a stronger evidence-base is required to guide decision making around biochemical monitoring.

These results should not be interpreted as representative of the opinions of all dog owners or veterinary professionals in the United Kingdom as the aim was to report the widest possible breadth of experience. However, as this area has not previously been explored using qualitative research, we highlight important issues and provide a foundation for future studies. It is possible that the method of owner recruitment introduced respondent bias in selecting owners who were highly committed to their dogs, but the range of interviewees and attitudes obtained was very broad. A failsafe formula does not exist to identify data saturation (Ziebland and McPherson, 2006) and there is always a risk that additional participants could have articulated previously unrepresented attitudes. However, additional themes did not emerge in either the final focus group or the last four interviews so it is likely that data saturation had occurred within the population available for inclusion. The inclusion criteria excluded owners whose dogs were untreated. Exploring the attitudes of those owners was outside the scope of this work but would be valuable future research. As with all qualitative research, analysis by others may have led to alternative themes. Coding was not replicated, as advocated by Morse (1997).

The findings of this study should be of value to anyone interested in improving analgesia in dogs with osteoarthritis. The significant barriers to compliance identified could be overcome by provision of more accurate and comprehensive data to both veterinary professionals and owners on the true frequency of adverse events associated with veterinary NSAIDs and how best to avoid them.

## Conflicts of interest

None of the authors declares any conflicts of interest relevant to this paper.
